# Association of Perioperative Teriparatide Use with Postoperative Complications in Spinal Fusion Surgery: A Systematic Review and Meta-Analysis

**DOI:** 10.3390/jcm15156088

**Published:** 2026-08-05

**Authors:** Norihiro Isogai, Haruki Funao, Ryo Mizukoshi, Keitaro Ito, Mitsuru Yagi

**Affiliations:** 1 Department of Orthopaedic Surgery, School of Medicine, International University of Health and Welfare, Chiba 286-8520, Japan; n.isogai0813@gmail.com (N.I.); hfunao@yahoo.co.jp (H.F.); masterplan.1651@gmail.com (R.M.); keitaro0518@gmail.com (K.I.); 2 Department of Orthopaedic Surgery, International University of Health and Welfare Narita Hospital, Chiba 286-8520, Japan

**Keywords:** teriparatide, spinal fusion surgery, meta-analysis, systematic review, complications, revision surgery, proximal junctional kyphosis

## Abstract

**Background/Objectives:** Teriparatide, a recombinant form of parathyroid hormone used for the treatment of osteoporosis, has also been investigated in patients undergoing spinal fusion surgery. The aim of this study was to evaluate the association between perioperative teriparatide use and postoperative complications after spinal fusion surgery through a meta-analysis of randomized controlled trials (RCTs) and observational studies. **Methods:** Systematic searches were conducted across PubMed, EMBASE, Cochrane Library, and Scopus for RCTs and observational studies that compared surgical outcomes in spinal instrumentation procedures with and without teriparatide. The primary pooled outcome was proximal junctional kyphosis (PJK). Secondary outcomes were radiographic nonunion, mechanical complications, reoperation rate, and incidence of new vertebral fractures. Meta-analyses were performed using Mantel–Haenszel models. **Results:** A total of 20 studies comprising 2543 patients with degenerative lumbar disease, adult spinal deformity, or osteoporotic vertebral fracture were included. Perioperative teriparatide use was associated with a lower incidence of PJK (Risk Ratio [RR] 0.40, 95% confidence interval [CI] 0.22–0.75, *p* = 0.004) and radiographic nonunion both on a per-patient basis (RR 0.66, 95% CI 0.47–0.93, *p* = 0.02) and on a per-level basis (RR 0.55, 95% CI 0.37–0.82, *p* = 0.004). Although there were no statistically significant differences in mechanical complications (RR 0.75, 95% CI 0.54–1.04, *p* = 0.08), reoperation rate (RR 0.76, 95% CI 0.53–1.08, *p* = 0.13), and new vertebral fractures (RR 0.60, 95% CI 0.31–1.17, *p* = 0.13), these outcomes numerically favored teriparatide. **Conclusions:** Perioperative teriparatide use was associated with lower rates of PJK and radiographic nonunion after spinal fusion surgery. However, the included studies were heterogeneous, and evidence for mechanical complications, reoperation, and new vertebral fractures remains limited.

## 1. Introduction

Teriparatide, a recombinant form of parathyroid hormone (PTH), is an anabolic treatment approved for osteoporosis [1]. In patients undergoing spinal fusion surgery, perioperative teriparatide has been investigated as an adjunctive osteoporosis therapy, and several meta-analyses have suggested potential benefits for selected postoperative outcomes [2,3]. However, findings remain inconsistent; for example, Pan et al. reported that teriparatide use was associated with improved fusion-related outcomes, whereas Cho et al. found no clear difference [3,4]. Moreover, complication rates after spine surgery are influenced by multiple factors, including diagnosis, surgical technique, and patient characteristics [5], and no clear consensus has been established regarding the association between teriparatide and postoperative complications. In addition, reoperation has not been sufficiently investigated as a pooled outcome. Therefore, the aim of this study was to investigate the association between perioperative teriparatide use and postoperative complications, including reoperation, after spinal fusion surgery through a meta-analysis of randomized controlled trials (RCTs) and observational studies.

## 2. Materials and Methods

The protocol for this systematic review was registered on PROSPERO (No. CRD42024547085). This systematic review and meta-analysis was conducted in accordance with the Preferred Reporting Items for Systematic Reviews and Meta-Analyses (Supplementary Materials PRISMA 2020 Checklist) guidelines and the predefined protocol [6,7,8].

### 2.1. Interventions

The intervention of interest for this systematic review was the perioperative administration of teriparatide in patients undergoing spinal fusion surgery. This encompasses any dosage and method of administration of teriparatide that is given during the perioperative period of the surgical procedure.

### 2.2. Outcomes

This systematic review and meta-analysis evaluated the following outcomes.

1. Incidence of junctional or adjacent segment pathology: We extracted data on proximal junctional kyphosis (PJK), distal junctional kyphosis (DJK), and adjacent segment disease when reported. Because only PJK data were available for pooling, the primary meta-analysis focused on PJK. Outcome ascertainment was based on each study’s radiographic definitions using plain radiographs, CT, or MRI.
2. Radiographic nonunion: Nonunion was defined as failure to achieve fusion according to each study’s imaging-based criteria at the reported follow-up time points.
3. Incidence of mechanical complications: Mechanical complications included hardware failure, screw loosening, cage subsidence, vertebral collapse or slippage, and related implant- or junction-related complications, as defined in each study and monitored during follow-up.
4. Reoperation rate: Reoperation was defined as any subsequent surgery required because of complications or failure after the index procedure, including nonunion, infection, mechanical failure, or other study-specific indications.
5. Incidence of new vertebral fractures: New vertebral fractures were identified using plain radiographs, CT, or MRI according to each study’s follow-up protocol.

### 2.3. Study Design

Eligibility criteria included RCTs and observational studies that compared treatment outcomes with and without teriparatide in spinal fusion surgery.

### 2.4. Inclusion Criteria

We included studies involving adult patients who underwent spinal fusion surgery.

### 2.5. Exclusion Criteria

Exclusion criteria were studies not reporting outcomes of the complications relevant to this review, and studies involving non-adult populations or non-spinal fusion surgeries including vertebroplasty and decompression surgery. Case reports, case series, and reviews were excluded, as these study designs are more prone to bias and provide a lower level of evidence.

### 2.6. Search Strategy

A systematic literature search was conducted in PubMed, EMBASE, Cochrane Library, and Scopus for studies published up to June 2026. Search terms included combinations of keywords such as “teriparatide”, “spinal fusion surgery”, “complications”, and “efficacy”. No language restrictions were applied. Reference lists of eligible articles were also manually reviewed to identify additional studies.

### 2.7. Trial Selection and Data Extraction

Two authors independently screened articles and abstracts to identify eligible studies and assessed the full texts of potentially relevant articles. Discrepancies were resolved through discussion or consultation with a third reviewer. Study characteristics were independently extracted by two authors, including publication year, study population, sample size, comparator interventions, treatment dosage, treatment indication, reported adverse events (name and category), and data relevant to risk of bias assessment.

### 2.8. Data Extraction and Statistical Analyses

We first analyzed the association between teriparatide use and junctional pathology; because only PJK data were available for pooling, the primary meta-analysis focused on PJK. Separate meta-analyses were then performed for radiographic nonunion, mechanical complications, reoperation rate, and new vertebral fractures. In all analyses, a *p*-value of <0.05 was considered statistically significant.

Two reviewers independently assessed the methodological quality of the included studies using Risk of Bias 2 (RoB2) for RCTs and ROBINS-I for non-randomized studies. Risk ratios (RRs) with 95% confidence intervals (CIs) were pooled using Mantel–Haenszel models. When necessary, a continuity correction of 0.5 was applied for zero-event cells. Fixed-effects models were used when heterogeneity was negligible; otherwise, random-effects models were applied. Heterogeneity was assessed using the I2 statistic and visual inspection of forest plots. All statistical analyses were performed using Review Manager (RevMan) Version 5.4 (The Cochrane Collaboration, Copenhagen, Denmark) and SPSS Statistics Version 29 (IBM Corp., Armonk, NY, USA).

### 2.9. Quality Assessment

Two reviewers independently evaluated the risk of bias in the included studies in accordance with the Cochrane Handbook for Systematic Reviews of Interventions (Version 6.1). For RCTs, we used the revised criteria incorporated in RevMan 5.4. For non-randomized studies, we applied the ROBINS-I tool (Risk Of Bias In Non-randomized Studies of Interventions).

## 3. Results

### 3.1. Literature Search

**Figure 1:**
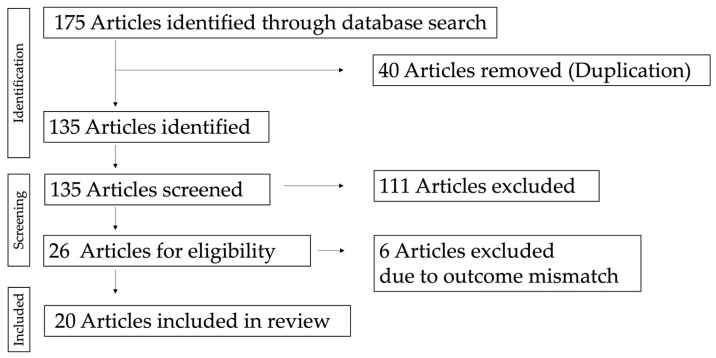
Preferred Reporting Items for Systematic Reviews and Meta-Analyses (PRISMA) flow diagram illustrating the study selection process.

In total, 175 records were identified, 40 duplicates were removed, and 135 titles/abstracts were screened. After full-text assessment of 26 articles, 20 studies involving 2543 participants were included in the meta-analysis [4,9,10,11,12,13,14,15,16,17,18,19,20,21,22,23,24,25,26,27] (Figure 1).

### 3.2. Description of Included Studies

Table 1 summarizes the characteristics of the included articles. Five articles were RCTs, five were prospective studies, and ten were retrospective studies. The diagnosis was degenerative lumbar disease in 13 articles, adult spinal deformity in four articles, osteoporotic vertebral fracture in two articles, and both degenerative lumbar disease and adult spinal deformity in one article. Surgical procedures were posterior lumbar interbody fusion or transforaminal lumbar interbody fusion in six articles, posterolateral lumbar fusion in six articles, adult spinal deformity surgery in four articles, posterior lumbar interbody fusion or lateral lumbar interbody fusion in one article, and any lumbar fusion surgery in three articles.

Preoperative femoral bone mineral density was reported in 15 studies. It was assessed using the T-score in seven studies, bone mineral density in seven studies, and young adult mean in six studies. In the comparison between the teriparatide and control groups, only one study showed a significant difference [18], whereas the remaining studies showed no significant differences (Table 2).

### 3.3. Risk of Bias in Individual Trials

**Figure 2:**
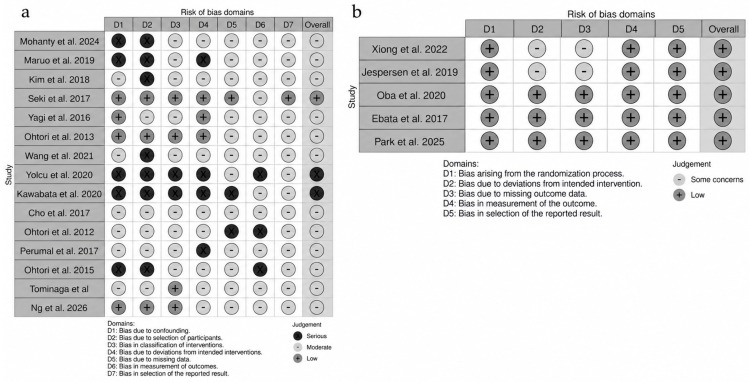
Risk-of-bias assessment. Several non-randomized studies showed moderate or serious risk of bias in selected domains (**a**) [4,9,10,12,14,15,16,17,18,19,21,22,24,26,27], whereas all included RCTs were judged to have a low risk of bias (**b**) [11,13,20,23,25].

Among the 5 RCTs, all were judged to have a low risk of bias. Among the 15 non-randomized studies, one study was judged to have a low risk of bias, 12 were judged to have a moderate risk of bias, and 2 were assessed as having a serious risk of bias in domains such as confounding, classification of interventions, selection of participants, and missing data (Figure 2).

### 3.4. Incidence of Proximal Junctional Kyphosis (Figure 3a)

**Figure 3:**
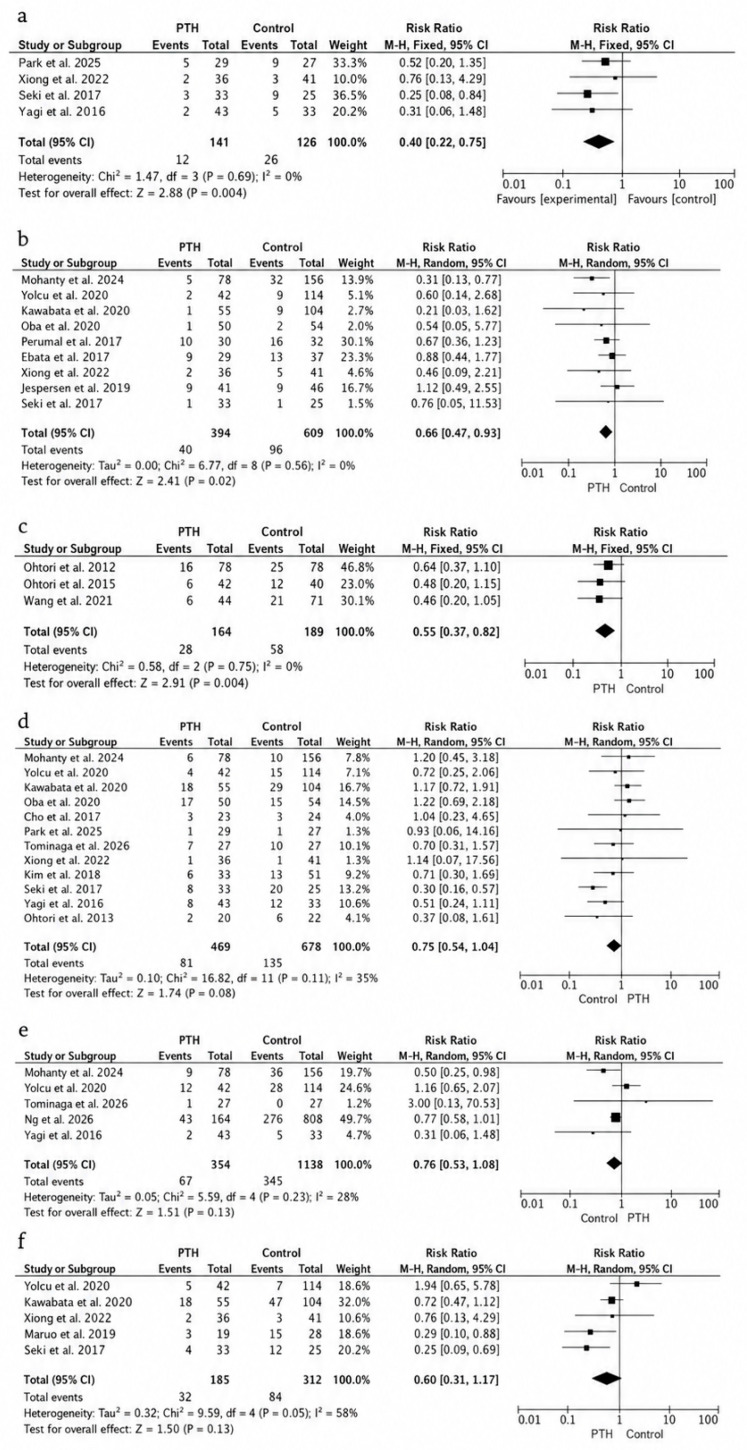
Forest plots of risk ratios for postoperative outcomes after spinal fusion surgery. (**a**). Proximal junctional kyphosis. (**b**). Radiographic nonunion (per-patients basis). (**c**). Radiographic nonunion (per-level basis). (**d**). Incidence of mechanical complications. (**e**). Reoperation rate. (**f**). Incidence of new vertebral fractures. Abbreviations: PTH, parathyroid hormone; CI, confidence [4,9,10,11,12,13,14,15,16,17,18,19,20,21,22,23,24,25,26,27].

Four studies analyzed PJK, whereas no studies reported poolable data for DJK. Of these, 1 focused on degenerative lumbar disease and 3 on adult spinal deformity. In the meta-analysis, teriparatide use was associated with a lower incidence of PJK (RR 0.40, 95% CI 0.22–0.75, *p* = 0.004) (Figure 3a). Among the individual studies, one demonstrated a statistically significant reduction [15], whereas the remaining three showed a non-significant trend favoring teriparatide [9,11,25]. The relative weight of each study was less than 40%.

### 3.5. Radiographic Nonunion (Figure 3b,c)

A total of 12 studies analyzed radiographic nonunion or fusion status. Of these, 7 focused on degenerative lumbar disease, 2 on adult spinal deformity, 2 on osteoporotic vertebral fractures, and 1 included both degenerative lumbar disease and adult spinal deformity. Nine studies assessed radiographic nonunion on a per-patient basis (Figure 3b), whereas three studies assessed radiographic nonunion on a per-level basis (Figure 3c). In the meta-analysis, teriparatide use was associated with a lower radiographic nonunion rate both on a per-patient basis (RR 0.66, 95% CI 0.47–0.93, *p* = 0.02) and on a per-level basis (RR 0.55, 95% CI 0.37–0.82, *p* = 0.004). Among the individual studies, only one study involving adult spinal deformity demonstrated a statistically significant difference [10], and the relative weight of the study was 13.9%.

### 3.6. Incidence of Mechanical Complications (Figure 3d)

A total of 12 studies analyzed mechanical complications after surgery. Of these, 6 focused on degenerative lumbar disease, 3 on adult spinal deformity, 2 on osteoporotic vertebral fractures, and 1 included both degenerative lumbar disease and adult spinal deformity. In the meta-analysis, teriparatide use was not significantly associated with a lower incidence of mechanical complications (RR 0.75, 95% CI 0.54–1.04, *p* = 0.08) (Figure 3d). Among the individual studies, a statistically significant difference was observed in one study involving adult spinal deformity [15], and the relative weight of the study was 13.2%.

### 3.7. Reoperation Rate (Figure 3e)

Five studies analyzed reoperation rate. Of these, 3 focused on degenerative lumbar disease and 2 on adult spinal deformity. Although one study reported a statistically significant difference [10], the pooled analysis showed that teriparatide use was not significantly associated with a lower reoperation rate (RR 0.76, 95% CI 0.53–1.08, *p* = 0.13) (Figure 3e).

### 3.8. Incidence of New Vertebral Fractures (Figure 3f)

A total of 5 studies analyzed new vertebral fractures after surgery. Of these, 2 focused on degenerative lumbar disease, 1 on adult spinal deformity, and 2 on osteoporotic vertebral fractures. Although two studies reported a statistically significant difference [12,15], the pooled analysis showed that teriparatide use was not significantly associated with a lower incidence of new vertebral fractures (RR 0.60, 95% CI 0.31–1.17, *p* = 0.13) (Figure 3f).

## 4. Discussion

In the present meta-analysis, perioperative teriparatide use was associated with lower rates of postoperative PJK, and radiographic nonunion. In contrast, no statistically significant differences were observed for mechanical complications, reoperation rate, or postoperative new vertebral fractures. Previous meta-analyses have reported broadly similar findings for nonunion-related and mechanical outcomes [3,28]. With respect to postoperative vertebral fractures, Pan et al. likewise reported no significant difference [3]. To our knowledge, reoperation has not previously been evaluated as a pooled outcome in this context, and the present findings therefore add clinically relevant information.

Previous studies examining the relationship between postoperative PJK and teriparatide have reported mixed results, with some demonstrating a beneficial association and others showing no significant difference [9,11,15]. Among the studies included in this meta-analysis, only one demonstrated a statistically significant effect when analyzed individually, whereas the other two showed only a trend favoring teriparatide. The pooled analysis nevertheless showed a significant association, suggesting that greater statistical power may have enabled detection of this relationship. PJK is a multifactorial complication, influenced not only by osteoporosis treatment but also by age, body mass index, bone quality, and surgical factors such as overcorrection of deformity and aggressive facetectomy at the upper instrumented vertebrae [29,30,31,32]. Therefore, small individual studies may fail to detect significant between-group differences.

Mechanical complications were not statistically significant; however, they tended to be less frequent in the teriparatide group in the present study. Previous reports have suggested that teriparatide use may be associated with reduced screw loosening and fewer complications within two years after surgery [33,34,35]. In patients with adult spinal deformity, preoperative osteoporosis management including teriparatide has been recommended in selected high-risk settings [36,37]. On the other hand, screw placement, smoking, frailty, global spinal alignment evaluated by the global alignment and proportion (GAP) score, and the patient demographics, radiographic index and surgical invasiveness for mechanical failure (PRISM) model have also been identified as risk factors for postoperative mechanical failure [38,39,40,41]. Accordingly, even if teriparatide contributes to risk reduction, comprehensive perioperative optimization remains essential.

The present study also showed a lower radiographic nonunion rate in patients receiving teriparatide. Mechanical failure and nonunion are closely related [42]; however, they are not identical, as failure can occur even in cases that ultimately achieve radiographic fusion. Accordingly, the present findings should be interpreted as showing an association with lower radiographic nonunion rather than as establishing a specific indication for bone-fusion promotion. Because the available studies remain limited and heterogeneous, further investigation of radiographic nonunion is warranted.

Regarding the incidence of new vertebral fractures, two studies reported a significantly lower incidence in the teriparatide group; however, the meta-analysis of five studies showed no statistically significant difference. Only the study by Yolcu et al. reported a higher incidence of new vertebral fractures in the teriparatide group than in the control group [18], which likely contributed substantially to the lack of a significant difference in the pooled analysis. Notably, this was the only included study in which the baseline characteristics differed between the two groups, with the teriparatide group having significantly lower preoperative bone mineral density (Table 2). Teriparatide is well known to reduce the risk of vertebral fractures through its anabolic effects on bone formation [1]. However, no clear threshold has been established regarding the severity of osteoporosis beyond which its fracture-preventive effect may be attenuated. Therefore, it is possible that the difference in preoperative bone mineral density reported by Yolcu et al. was large enough that the beneficial effect of teriparatide could not fully compensate for the higher baseline fracture risk.

Although the reoperation rate numerically favored teriparatide, the difference was not statistically significant. To our knowledge, no previous meta-analysis has specifically evaluated reoperation after spine surgery in relation to teriparatide use. Lower rates of nonunion and mechanical failure may plausibly contribute to a lower risk of reoperation [38,43]. However, reoperation after spine surgery arises from diverse causes, including implant-related problems, infection, adjacent segment disease, residual stenosis, and implant malposition [44,45,46]. In particular, reoperations for adjacent segment disease often occur more than five years after surgery [47], and these events were not adequately captured in the present analysis. In addition, the included studies were heterogeneous with respect to baseline osteoporosis severity, alignment parameters, smoking status, and other clinical variables. Therefore, larger studies with longer follow-up and more standardized reporting are needed to clarify the association between teriparatide and reoperation.

There were several limitations in this study. First, there was substantial clinical heterogeneity among the included studies in terms of patient characteristics, diagnosis, surgical procedures, comparator treatments, and teriparatide protocols. Especially, the diagnosis included degenerative lumbar disease, adult spinal deformity, and osteoporotic vertebral fracture, which differ in pathophysiology, surgical invasiveness, fusion length, and complication profiles. Therefore, although a random-effects model was used, the pooled estimates should be interpreted cautiously, and the findings should be considered exploratory rather than definitive. Second, important confounding factors such as bone mineral density, age, and surgical alignment were not fully controlled across studies. In particular, preoperative bone mineral density was not measured in five studies, and the assessment methods were not standardized across studies. Therefore, direct comparison among studies or pooled analysis was not feasible. Third, the sample size of each study was relatively small, which may limit statistical power. In particular, the analyses of PJK and reoperation rate were based on only four and five studies, respectively; therefore, further studies are needed to validate these findings. Fourth, definitions and units of analysis for radiographic and mechanical outcomes were not completely uniform across studies, which may have introduced additional heterogeneity. Fifth, publication bias could not be excluded. Sixth, most of the included non-randomized studies had at least moderate risk of bias. Although no analysis showed an extreme imbalance in which the relative weight was concentrated in a single study, non-randomized studies contributed to the overall estimates together with randomized controlled trials in the pooled analysis; therefore, the results may have been influenced by the methodological quality of the included observational studies. Seventh, the dose, duration, and timing of teriparatide treatment were not consistently reported, which precluded subgroup analyses based on these factors.

Despite these limitations, this meta-analysis provides an overview of the available comparative evidence regarding perioperative teriparatide use and postoperative outcomes after spinal fusion surgery.

## 5. Conclusions

The present findings suggest that perioperative teriparatide use is associated with lower rates of PJK and radiographic nonunion after spinal fusion surgery. In contrast, evidence regarding mechanical complications, reoperation, and new vertebral fractures remains limited, and these outcomes warrant further investigation.

## Figures and Tables

**Table 1:** Summary of the Characteristics of the Included Articles.

Study ID	Authors	Study Design	Country	Diagnosis	Type of Surgery	Sample Size		Mean Age	
						Teriparatide	Control	Teriparatide	Control
1	Mohanty et al. 2024 [10]	Retrospective	USA	ASD	ASD	78	156	68.5 ± 7.1	68.8 ± 8.4
2	Xiong et al. 2022 [11]	RCT	China	Degenerative	PLIF	36	41	63.1 ± 7.4	63.6 ± 8.3
3	Maruo et al. 2019 [12]	Retrospective	Japan	OVF	PLF	19	28	75.6 ± 5.7	75.5 ± 6.9
4	Jespersen et al. 2019 [13]	RCT	Denmark	Degenerative	PLF	41	46	71.0 ± 1.0	70.0 ± 0.9
5	Kim et al. 2018 [14]	Retrospective	Korea	Degenerative	TLIF	33	51	69.5 ± 7.8	66.8 ± 6.5
6	Seki et al. 2017 [15]	Prospective	Japan	ASD	ASD	33	25	72.5 ± 5.0	71.5 ± 2.0
7	Yagi et al. 2016 [9]	Prospective	Japan	ASD	ASD	43	33	68.6 ± 6.9	66.7 ± 6.9
8	Ohtori et al. 2013 [16]	Prospective	Japan	Degenerative	PLIF	20	22	78.0 ± 6.0	77.0 ± 5.8
9	Wang et al. 2021 [17]	Retrospective	China	Degenerative	TLIF	29	38	66.3 ± 8.1	65.9 ± 6.1
10	Yolcu et al. 2020 [18]	Retrospective	USA	Degenerative or ASD	Fusion Surgery	42	114	62.0 ± 6.0	70.0 ± 6.0
11	Kawabata et al. 2020 [19]	Retrospective	Japan	OVF	PLF	55	104	76.4 ± 6.4	75.2 ± 6.8
12	Oba et al. 2020 [20]	RCT	Japan	Degenerative or ASD	PLIF or LLIF	50	54	72.5 ± 7.3	72.0 ± 7.5
13	Cho et al. 2017 [4]	Prospective	Korea	Degenerative	PLIF	23	24	71.0 ± 4.9	68.2 ± 8.4
14	Ohtori et al. 2012 [21]	Prospective	Japan	Degenerative	PLF	29	28	76.0 ± 6.5	77.0 ± 6.0
15	Perumal et al. 2017 [22]	Retrospective	Taiwan	Degenerative	PLF	30	32	69.8 ± 3.8	70.4 ± 3.6
16	Ebata et al. 2017 [23]	RCT	Japan	Degenerative	PLIF	36	38	72.6 ± 7.0	70.4 ± 8.0
17	Ohtori et al. 2015 [24]	Retrospective	Japan	Degenerative	PLF	15	15	72.0 ± 7.7	71.0 ± 6.0
18	Park et al. 2025 [25]	RCT	Korea	ASD	ASD	29	27	70.9 ± 12.8	71.7 ± 7.4
19	Tominaga et al. 2026 [26]	Retrospective	Japan	Degenerative	Fusion Surgery	27	27	75 (71.5–79)	74 (66–78.5)
20	Ng et al. 2026 [27]	Retrospective	USA	Degenerative	Fusion Surgery	164	808	63.3 ± 8.5	63.4 ± 8.5

Abbreviations: RCT, randomized controlled trial; USA, United States of America; ASD, adult spinal deformity; OVF, osteoporotic vertebral fracture; PLIF, posterior lumbar interbody fusion; PLF, posterolateral lumbar fusion; TLIF, transforaminal lumbar interbody fusion; LLIF, lateral lumbar interbody fusion. Mean and standard deviation. For Study 19, the standard deviation was not reported; therefore, the range is presented instead.

**Table 2:** Baseline Hip Bone Mineral Density of the Included Articles.

Study ID	Authors	T Score		BMD (g/cm^2^)		YAM (%)	
		Teriparatide	Control	Teriparatide	Control	Teriparatide	Control
1	Mohanty et al. 2024 [10]	−2.74	−1.48	_	_	_	_
2	Xiong et al. 2022 [11]	_	_	0.492	0.504	_	_
3	Maruo et al. 2019 [12]	_	_	_	_	_	_
4	Jespersen et al. 2019 [13]	_	_	_	_	_	_
5	Kim et al. 2018 [14]	−3.09	−2.94	_	_	_	_
6	Seki et al. 2017 [15]	−3.9	−3.8	0.652	0.677	_	_
7	Yagi et al. 2016 [9]	_	_	0.771	0.729	_	_
8	Ohtori et al. 2013 [16]	_	_	_	_	66	64
9	Wang et al. 2021 [17]	_	_	_	_	_	_
10	Yolcu et al. 2020 [18]	_	_	0.72 *	0.82	_	_
11	Kawabata et al. 2020 [19]	_	_	_	_	73	72.5
12	Oba et al. 2020 [20]	_	_	0.65	0.67	73.7	76.5
13	Cho et al. 2017 [4]	−3.7	−3.5	_	_	_	_
14	Ohtori et al. 2012 [21]	_	_	_	_	65	64
15	Perumal et al. 2017 [22]	_	_	_	_	_	_
16	Ebata et al. 2017 [23]	−2.39	−2.27	_	_	72.3	73.6
17	Ohtori et al. 2015 [24]	_	_	_	_	65.6	66.8
18	Park et al. 2025 [25]	−3	−2.9	0.651	0.569	_	_
19	Tominaga et al. 2026 [26]	−2.3	−2.7	0.7	0.65	_	_
20	Ng et al. 2026 [27]	_	_	_	_	_	_

Abbreviations: BMD, bone mineral density; YAM, young adult mean. *: Significant differences compared to the control group (*p* < 0.05).

## Data Availability

The data supporting the findings of this study are not publicly available but are available from the corresponding author upon reasonable request.

## Supplementary Materials

The following supporting information can be downloaded at: https://www.mdpi.com/article/10.3390/jcm15156088/s1, PRISMA 2020 Checklist.

## Abbreviations

The following abbreviations are used in this manuscript:

PTH	parathyroid hormone
PJK	Proximal junctional kyphosis
DJK	Distal junctional kyphosis
RCT	Randomized controlled trial
USA	United States of America
ASD	Adult spinal deformity
OVF	Osteoporotic vertebral fracture
PLIF	Posterior lumbar interbody fusion
PLF	Posterolateral lumbar fusion
TLIF	Transforaminal lumbar interbody fusion
LLIF	Lateral lumbar interbody fusion

## References

[1] Eastell R., Walsh J.S. (2017). Anabolic treatment for osteoporosis: Teriparatide. Clin. Cases Min. Bone Metab..

[2] Cheng S.H., Kuo Y.J., Chen C., Kang Y.N. (2020). Effects of teriparatide and bisphosphonate on spinal fusion procedure: A systematic review and network meta-analysis. PLoS ONE.

[3] Pan T.Y., Chang C.C., Chen H.T., Tsou H.K., Lin Y.C., Hsu C.H. (2023). Effectiveness of Teriparatide for Spine Fusion in Osteoporotic Patient: A Systematic Review and Meta-Analysis of Comparative Studies. World Neurosurg..

[4] Cho P.G., Ji G.Y., Shin D.A., Ha Y., Yoon D.H., Kim K.N. (2017). An effect comparison of teriparatide and bisphosphonate on posterior lumbar interbody fusion in patients with osteoporosis: A prospective cohort study and preliminary data. Eur. Spine J..

[5] Lee M.J., Konodi M.A., Cizik A.M., Bransford R.J., Bellabarba C., Chapman J.R. (2012). Risk factors for medical complication after spine surgery: A multivariate analysis of 1591 patients. Spine J..

[6] Higgins J. (2008). Cochrane Handbook for Systematic Reviews of Interventions.

[7] Jakobsen J.C., Wetterslev J., Winkel P., Lange T., Gluud C. (2014). Thresholds for statistical and clinical significance in systematic reviews with meta-analytic methods. BMC Med. Res. Methodol..

[8] Page M.J., McKenzie J.E., Bossuyt P.M., Boutron I., Hoffmann T.C., Mulrow C.D., Shamseer L., Tetzlaff J.M., Akl E.A., Brennan S.E. (2021). The PRISMA 2020 statement: An updated guideline for reporting systematic reviews. BMJ.

[9] Yagi M., Ohne H., Konomi T., Fujiyoshi K., Kaneko S., Komiyama T., Takemitsu M., Yato Y., Machida M., Asazuma T. (2016). Teriparatide improves volumetric bone mineral density and fine bone structure in the UIV+1 vertebra, and reduces bone failure type PJK after surgery for adult spinal deformity. Osteoporos. Int..

[10] Mohanty S., Sardar Z.M., Hassan F.M., Lombardi J.M., Lehman R.A., Lenke L.G. (2024). Impact of Teriparatide on Complications and Patient-Reported Outcomes of Patients Undergoing Long Spinal Fusion According to Bone Density. J. Bone Jt. Surg. Am..

[11] Xiong Y., Li L., Liu P., Zhou B., Kang Y., Wang G. (2022). Effect of Teriparatide Versus Zoledronate on Posterior Lumbar Interbody Fusion in Postmenopausal Women with Osteoporosis. World Neurosurg..

[12] Maruo K., Tachibana T., Arizumi F., Kusuyama K., Kishima K., Yoshiya S. (2019). Effect of Teriparatide on Subsequent Vertebral Fractures after Instrumented Fusion Surgery for Osteoporotic Vertebral Fractures with Neurological Deficits. Asian Spine J..

[13] Jespersen A.B., Andresen A.D.K., Jacobsen M.K., Andersen M.O., Carreon L.Y. (2019). Does Systemic Administration of Parathyroid Hormone After Noninstrumented Spinal Fusion Surgery Improve Fusion Rates and Fusion Mass in Elderly Patients Compared to Placebo in Patients with Degenerative Lumbar Spondylolisthesis?. Spine.

[14] Kim J.W., Park S.W., Kim Y.B., Ko M.J. (2018). The Effect of Postoperative Use of Teriparatide Reducing Screw Loosening in Osteoporotic Patients. J. Korean Neurosurg. Soc..

[15] Seki S., Hirano N., Kawaguchi Y., Nakano M., Yasuda T., Suzuki K., Watanabe K., Makino H., Kanamori M., Kimura T. (2017). Teriparatide versus low-dose bisphosphonates before and after surgery for adult spinal deformity in female Japanese patients with osteoporosis. Eur. Spine J..

[16] Ohtori S., Inoue G., Orita S., Yamauchi K., Eguchi Y., Ochiai N., Kishida S., Kuniyoshi K., Aoki Y., Nakamura J. (2013). Comparison of teriparatide and bisphosphonate treatment to reduce pedicle screw loosening after lumbar spinal fusion surgery in postmenopausal women with osteoporosis from a bone quality perspective. Spine.

[17] Wang Z., Zhuang C., Chen W., Li Z., Li J., Lin H., Jian D. (2021). The Effect of Daily Teriparatide versus One-Time Annually Zoledronic Acid Administration After Transforaminal Lumbar Interbody Fusion in Osteoporotic Patients. Clin. Interv. Aging.

[18] Yolcu Y.U., Zreik J., Alvi M.A., Wanderman N.R., Carlson B.C., Nassr A., Fogelson J.L., Elder B.D., Freedman B.A., Bydon M. (2020). Use of teriparatide prior to lumbar fusion surgery lowers two-year complications for patients with poor bone health. Clin. Neurol. Neurosurg..

[19] Kawabata A., Yoshii T., Hirai T., Ushio S., Kaito T., Yamashita T., Fujiwara H., Nagamoto Y., Matsuoka Y., Suzuki H. (2020). Effect of bisphosphonates or teriparatide on mechanical complications after posterior instrumented fusion for osteoporotic vertebral fracture: A multi-center retrospective study. BMC Musculoskelet. Disord..

[20] Oba H., Takahashi J., Yokomichi H., Hasegawa T., Ebata S., Mukaiyama K., Ohba T., Ushirozako H., Kuraishi S., Ikegami S. (2020). Weekly Teriparatide Versus Bisphosphonate for Bone Union During 6 Months After Multi-Level Lumbar Interbody Fusion for Osteoporotic Patients: A Multicenter, Prospective, Randomized Study. Spine.

[21] Ohtori S., Inoue G., Orita S., Yamauchi K., Eguchi Y., Ochiai N., Kishida S., Kuniyoshi K., Aoki Y., Nakamura J. (2012). Teriparatide accelerates lumbar posterolateral fusion in women with postmenopausal osteoporosis: Prospective study. Spine.

[22] Kaliya-Perumal A.K., Lu M.L., Luo C.A., Tsai T.T., Lai P.L., Chen L.H., Chen W.J., Niu C.C. (2017). Retrospective radiological outcome analysis following teriparatide use in elderly patients undergoing multilevel instrumented lumbar fusion surgery. Medicine.

[23] Ebata S., Takahashi J., Hasegawa T., Mukaiyama K., Isogai Y., Ohba T., Shibata Y., Ojima T., Yamagata Z., Matsuyama Y. (2017). Role of Weekly Teriparatide Administration in Osseous Union Enhancement within Six Months After Posterior or Transforaminal Lumbar Interbody Fusion for Osteoporosis-Associated Lumbar Degenerative Disorders: A Multicenter, Prospective Randomized Study. J. Bone Jt. Surg. Am..

[24] Ohtori S., Orita S., Yamauchi K., Eguchi Y., Ochiai N., Kuniyoshi K., Aoki Y., Nakamura J., Miyagi M., Suzuki M. (2015). More than 6 Months of Teriparatide Treatment Was More Effective for Bone Union than Shorter Treatment Following Lumbar Posterolateral Fusion Surgery. Asian Spine J..

[25] Park J.H., Kwon O., Choi J.H., Yeom J.S., Park S.M., Kim C.H., Kim H.J. (2025). Perioperative teriparatide for preventing proximal junctional kyphosis and failure in patients with osteoporosis after adult thoracolumbar spinal deformity surgery: A prospective randomized controlled trial. Osteoporos. Int..

[26] Tominaga A., Wada K., Okazaki K. (2025). Vertebral changes evaluated by hounsfield units after spinal fusion surgery with concomitant anabolic therapy. Arch. Osteoporos..

[27] Ng M.K., Rodriguez A.N., Razi A., Emara A.K., Ford B.T., Tabbaa A., Gouni D., Xu J.J., Mastrokostas P.G., Monsef J.B. (2026). Teriparatide use in osteopenic patients undergoing single-level lumbar fusion associated with decreased 2-year revision rates. J. Craniovertebr. Junction Spine.

[28] Buerba R.A., Sharma A., Ziino C., Arzeno A., Ajiboye R.M. (2018). Bisphosphonate and Teriparatide Use in Thoracolumbar Spinal Fusion: A Systematic Review and Meta-analysis of Comparative Studies. Spine.

[29] Yagi M., Akilah K.B., Boachie-Adjei O. (2011). Incidence, risk factors and classification of proximal junctional kyphosis: Surgical outcomes review of adult idiopathic scoliosis. Spine.

[30] Yagi M., Fujita N., Tsuji O., Nagoshi N., Asazuma T., Ishii K., Nakamura M., Matsumoto M., Watanabe K. (2018). Low Bone-Mineral Density Is a Significant Risk for Proximal Junctional Failure After Surgical Correction of Adult Spinal Deformity: A Propensity Score-Matched Analysis. Spine.

[31] Yagi M., Yamanouchi K., Fujita N., Funao H., Ebata S. (2023). Proximal Junctional Failure in Adult Spinal Deformity Surgery: An In-depth Review. Neurospine.

[32] Chen J.W., McCandless M.G., Bhandarkar A.R., Flanigan P.M., Lakomkin N., Mikula A.L., Michalopoulos G.D., Bydon M. (2023). The association between bone mineral density and proximal junctional kyphosis in adult spinal deformity: A systematic review and meta-analysis. J. Neurosurg. Spine.

[33] Tsai S.H.L., Chien R.S., Lichter K., Alharthy R., Alvi M.A., Goyal A., Bydon M., Fu T.S., Lin T.Y. (2020). Teriparatide and bisphosphonate use in osteoporotic spinal fusion patients: A systematic review and meta-analysis. Arch. Osteoporos..

[34] Shibuya Y., Katsumi K., Ohashi M., Tashi H., Makino T., Yamazaki A., Hirano T., Sawakami K., Kikuchi R., Kawashima H. (2022). Effect of adjuvant therapy with teriparatide in patients with thoracolumbar osteoporotic vertebral fractures who underwent vertebroplasty with posterior spinal fusion. Sci. Rep..

[35] Inoue G., Ueno M., Nakazawa T., Imura T., Saito W., Uchida K., Ohtori S., Toyone T., Takahira N., Takaso M. (2014). Teriparatide increases the insertional torque of pedicle screws during fusion surgery in patients with postmenopausal osteoporosis. J. Neurosurg. Spine.

[36] Raad M., Ortiz-Babilonia C., Hassanzadeh H., Puvanesarajah V., Kebaish K., Jain A. (2022). Cost-utility Analysis of Neoadjuvant Teriparatide Therapy in Osteopenic Patients Undergoing Adult Spinal Deformity Surgery. Spine.

[37] Sardar Z.M., Coury J.R., Cerpa M., DeWald C.J., Ames C.P., Shuhart C., Watkins C., Polly D.W., Dirschl D.R., Klineberg E.O. (2022). Best Practice Guidelines for Assessment and Management of Osteoporosis in Adult Patients Undergoing Elective Spinal Reconstruction. Spine.

[38] Inoue S., Khashan M., Fujimori T., Berven S.H. (2015). Analysis of mechanical failure associated with reoperation in spinal fusion to the sacrum in adult spinal deformity. J. Orthop. Sci..

[39] Yilgor C., Sogunmez N., Boissiere L., Yavuz Y., Obeid I., Kleinstuck F., Pérez-Grueso F.J.S., Acaroglu E., Haddad S., Mannion A.F. (2017). Global Alignment and Proportion (GAP) Score: Development and Validation of a New Method of Analyzing Spinopelvic Alignment to Predict Mechanical Complications After Adult Spinal Deformity Surgery. J. Bone Jt. Surg. Am..

[40] Williams J.L., Sarraj M., Mechas C.A., Pennington Z., Pumford A.D., Postal Z., Tucker J., Helgeson M.D., Huddleston P.M., Nassr A.N. (2025). The Impact of Screw Density on Mechanical Complications in Lumbar Spine Deformity Constructs. Spine.

[41] Yagi M., Hosogane N., Fujita N., Okada E., Suzuki S., Tsuji O., Nagoshi N., Nakamura M., Matsumoto M., Watanabe K. (2020). The patient demographics, radiographic index and surgical invasiveness for mechanical failure (PRISM) model established for adult spinal deformity surgery. Sci. Rep..

[42] Park S.J., Park J.S., Lee C.S., Kang D.H. (2025). Risk Factors for Rod Fracture. J. Clin. Med..

[43] Grotle M., Smastuen M.C., Fjeld O., Grovle L., Helgeland J., Storheim K., Solberg T.K., Zwart J.A. (2019). Lumbar spine surgery across 15 years: Trends, complications and reoperations in a longitudinal observational study from Norway. BMJ Open.

[44] Taniguchi Y., Ohara T., Suzuki S., Watanabe K., Suzuki T., Uno K., Yamaguchi T., Yanagida H., Nakayama K., Kotani T. (2021). Incidence and Risk Factors for Unplanned Return to the Operating Room Following Primary Definitive Fusion for Pediatric Spinal Deformity: A Multicenter Study with Minimum 2-year Follow-Up. Spine.

[45] Hilibrand A.S., Robbins M. (2004). Adjacent segment degeneration and adjacent segment disease: The consequences of spinal fusion?. Spine J..

[46] Martin B.I., Mirza S.K., Comstock B.A., Gray D.T., Kreuter W., Deyo R.A. (2007). Reoperation rates following lumbar spine surgery and the influence of spinal fusion procedures. Spine.

[47] Nakashima H., Kawakami N., Tsuji T., Ohara T., Suzuki Y., Saito T., Nohara A., Tauchi R.M., Ohta K., Hamajima N. (2015). Adjacent Segment Disease After Posterior Lumbar Interbody Fusion: Based on Cases with a Minimum of 10 Years of Follow-up. Spine.

